# Progression to complete retinal pigment epithelium and outer retinal atrophy (cRORA): post hoc analysis of the GATHER1 trial

**DOI:** 10.1007/s00417-024-06676-7

**Published:** 2024-11-14

**Authors:** Giulia Corradetti, Ayesha Karamat, Sowmya Srinivas, Sophiana Lindenberg, Swetha B. Velaga, Federico Corvi, Yamini Attiku, Muneeswar Gupta Nittala, Dhaval Desai, Liansheng Zhu, Dina Abulon, SriniVas R. Sadda

**Affiliations:** 1 Doheny Image Reading and Research Lab, Doheny Eye Institute, 150 N. Orange Grove Blvd, Pasadena, CA 91103 USA; 2 Formerly Astellas Pharma Global Development, Inc., Northbrook, IL USA; 3 Astellas Pharma US, Northbrook, IL USA; 4 Department of Ophthalmology, David Geffen School of Medicine at the University of California, Los Angeles, Los Angeles, CA USA

**Keywords:** Age-related macular degeneration, Geographic atrophy, Avacincaptad pegol, Drusen

## Abstract

**Purpose:**

Determine rates of progression of incomplete retinal pigment epithelium and outer retinal atrophy (iRORA) to complete retinal pigment epithelium and outer retinal atrophy (cRORA) and rates of progression of drusen to iRORA/cRORA in eyes with geographic atrophy (GA) treated with avacincaptad pegol (ACP).

**Methods:**

Post hoc analysis of the GATHER1 prospective, randomized, double-masked Phase II/III study that evaluated ACP 2 mg vs. sham. Optical coherence tomography (OCT) data from GATHER1 were transferred to the Doheny Image Reading and Research Lab for masked analysis by readers experienced with Classification of Atrophy Meeting (CAM) grading features. Regions of OCT volume scans more than 500 µm from the border of GA lesions were evaluated at baseline and at months 6, 12, and 18. Participants with iRORA and/or drusen (≥ 40 µm height on OCT) at baseline were included in the analysis.

**Results:**

The proportion of eyes progressing from iRORA to cRORA in the ACP 2 mg group was 5.0%, 15.0%, and 20.0% at months 6, 12, and 18 respectively, as compared with 11.8%, 30.2%, and 41.8% of eyes in the sham group. The proportion of ACP 2 mg-treated eyes progressing from drusen to iRORA or cRORA was 3.8%, 7.6%, and 7.6% at months 6, 12, and 18 compared with 15.9%, 18.1%, and 27.2% of sham-treated eyes.

**Conclusions:**

Rates of progression from iRORA to cRORA and drusen to iRORA/cRORA were reduced in eyes treated with ACP 2 mg vs. sham, with increasing separation between groups over time, suggesting early intervention may slow disease progression.

**Trial registration:**

ClinicalTrials.gov identifier: NCT02686658. Date of registration: February 16, 2016.

**Key messages:**

***What is known***

- Geographic atrophy is an advanced form of age-related macular degeneration (AMD) that leads to irreversible vision loss, presenting a significant public health unmet need.
- The Classification of Atrophy Meeting (CAM) group recommended a new nomenclature for advanced AMD lesions, based on the affected anatomical layers on optical coherence tomography. Accordingly, the terms incomplete retinal pigment epithelium and outer retinal atrophy (iRORA) and complete retinal pigment epithelium and outer retinal atrophy (cRORA) were introduced (Guymer et al., Ophthalmology 127:394–409, 2020; Sadda et al., Ophthalmology 125:537–548, 2018).

***What is new***

- GATHER1 post hoc analysis shows that treatment with avacincaptad pegol (ACP) 2 mg decreases the proportion of eyes that progress from iRORA to cRORA, and from drusen to iRORA or cRORA, compared with sham, over 6, 12, and 18 months.
- These findings suggest a potential role for ACP in delaying the progression of existing pre-atrophic AMD lesions

## Introduction

Age-related macular degeneration (AMD) is a progressive degenerative disease of the retina that can lead to irreversible vision loss [1, 2]. The hallmark of early AMD is the presence of drusen, extracellular deposits of lipid and protein debris, between the retinal pigment epithelium (RPE) and Bruch’s membrane [1–4]. The Beckman classification notes that early AMD is characterized by medium drusen (> 63 µm and ≤ 125 µm in diameter) and no AMD pigmentary abnormalities, and intermediate AMD consists of large drusen (> 125 µm in diameter) and any AMD hyper- or hypopigmentation abnormalities. Late AMD is defined as neovascular AMD (wet AMD) and/or any geographic atrophy (dry AMD) [4]. Geographic atrophy (GA) is a degenerative disease marked by the progressive loss of retinal photoreceptors, RPE, and underlying choriocapillaris, leading to irreversible vision loss [1, 3]. Irreversible loss of visual function due to GA greatly affects patients’ quality of life, as it results in loss of independence and mobility [5–7]. To date, there are only 2 treatments for GA approved by the US Food and Drug Administration (FDA) [8, 9]. Therefore, GA represents a significant public health unmet need.

The pathophysiology of GA has not been completely elucidated. However, several lines of evidence, including genetic, immunohistologic, and preclinical findings, link malfunction of the complement system to AMD and GA. Furthermore, complement activation products have been found in the blood and eyes of patients with advanced AMD postmortem. Inhibition of the complement pathway is therefore a potential therapeutic strategy for slowing GA progression [3, 10, 11].

Avacincaptad pegol (ACP), a pegylated ribonucleic acid (RNA) aptamer, is a specific inhibitor of complement component 5 (C5). ACP inhibits cleavage of C5, the key terminal effector of the complement system regardless of the initiation pathway, preventing inflammation and cell death while preserving the immunomodulatory effects of earlier complement components [12, 13]. In the GATHER1 prospective, randomized, double-masked, sham-controlled Phase II/III study (NCT02686658), both ACP 2 mg and 4 mg met their prespecified primary efficacy objectives, demonstrating statistically significant reductions in mean GA growth rate over 12 months vs. sham, as measured by fundus autofluorescence (FAF) [13]. The reduction in mean GA growth (square root transformed) over 12 months was 27.4% (difference: 0.110 mm; 95% confidence interval [CI]: 0.030–0.190; *p* = 0.0072) for ACP 2 mg compared with its corresponding sham and 27.8% (difference: 0.124 mm; 95% CI: 0.038–0.209; *p* = 0.0051) for ACP 4 mg compared with its corresponding sham [13]. Over 18 months, ACP was associated with continued reduction in GA lesion growth vs. sham [14]. ACP was generally well tolerated over 12 and 18 months, and the most frequently reported ocular adverse events were related to the injection procedure [13, 14].

Color fundus photography (CFP) and FAF have been the main imaging methods used to assess atrophy growth in clinical trials [15, 16]. However, the ability to identify anatomic biomarkers that predict GA development to determine if a treatment can reduce the progression to GA would be an important addition to clinical trials and in real-world settings. The Classification of Atrophy Meeting (CAM) group recommended optical coherence tomography (OCT) as the primary modality to identify early atrophic stages of AMD. The high resolution of OCT allows visualization of distinct retinal layers to detect early AMD lesions before they become detectable by CFP or FAF, and identification of risk factors associated with AMD progression when used with other imaging modalities [16, 17]. Moreover, OCT technology is widely available and noninvasive [16, 18, 19]. CAM recommended a new nomenclature for advanced AMD lesions based on the affected anatomic layers on OCT, introducing the terms incomplete retinal pigment epithelium and outer retinal atrophy (iRORA) and complete retinal pigment epithelium and outer retinal atrophy (cRORA) [15, 16].

cRORA is defined by the following criteria: (1) a region of hypertransmission of ≥ 250 μm in diameter, and (2) a zone of attenuation or disruption of the RPE of ≥ 250 μm in diameter, and (3) evidence of overlying photoreceptor degeneration, all occurring in the absence of signs of an RPE tear [15, 16]. The term iRORA describes a stage of AMD in which these OCT signs are present but do not fulfill all the criteria for cRORA and should also not be used in the presence of an RPE tear. It is defined on OCT by the following criteria: (1) a region of signal hypertransmission into the choroid, and (2) a corresponding zone of attenuation or disruption of the RPE < 250 μm, with or without persistence of basal laminar deposits, and (3) evidence of overlying photoreceptor degeneration. iRORA can progress to cRORA within a range of months to years [15].

This post hoc analysis was conducted to better understand the impact of ACP treatment on the progression from iRORA to cRORA and from drusen to iRORA and/or cRORA in eyes with GA.

## Methods

### Study design

This was a post hoc analysis of GATHER1, a prospective, randomized, double-masked, multicenter Phase II/III trial that evaluated the efficacy and safety of ACP administered monthly by intravitreal injection. Participants (*N* = 286) with non−center point-involving GA with lesions, in part, within 1500 µm from the center of the fovea, were enrolled at 63 centers in the United States, Europe, and Israel. The trial is registered at ClinicalTrials.gov (NCT02686658), and detailed methodology has previously been published [13].

Briefly, GATHER1 consisted of 2 parts: In Part 1, patients were randomized in a 1:1:1 ratio to the following dose groups: ACP 1 mg, ACP 2 mg, and sham. Subsequently, in Part 2, patients were randomized in a 1:2:2 ratio to the following dose groups: ACP 2 mg, ACP 4 mg, and sham. ACP or sham was administered monthly for 18 months.

Participants in the ACP 2 mg and sham groups were included in this analysis if they received at least one dose of study drug (intent-to-treat [ITT] population), and had gradable baseline and follow-up images and the presence of iRORA and/or drusen of at least 40 μm in height at baseline. Drusen height has been described as a risk factor for progression to advanced AMD [20]. Patients were excluded if they developed macular neovascularization (MNV), only had screening images, or had an early termination visit.

The GATHER1 trial was performed in accordance with the tenets of the Declaration of Helsinki and the International Conference on Harmonisation Good Clinical Practice guidelines. Written informed consent was obtained from all participants [13].

### Image acquisition

In GATHER1, GA progression was evaluated based on the change in GA lesion area over 6, 12, and 18 months. Using the modified 3-field imaging protocol (field 1 M: 30-degree field centered on the temporal aspect of the optic nerve, field 2: 30-degree field centered on the foveal center, and field 3 M: 30-degree field centered at a location 1–1.5 disc diameters temporal to the center of field 2), blue light FAF, color fundus photographs, and fluorescein angiograms were obtained. A Heidelberg Spectralis or HRA (Heidelberg Engineering, Heidelberg, Germany) system was used to capture FAF and near-infrared field 2 images. Spectral-domain OCT scans were obtained with the Cirrus (Carl Zeiss Meditec, Jena, Germany) or Heidelberg Spectralis systems. Spectralis scans were obtained with 97-line volume scan (20⁰ × 20⁰, high-resolution mode, Automatic Real-Time [ART] = 9) and 73-line volume scan (20⁰ × 15⁰, high-resolution mode, ART = 9) protocols. Cirrus OCT scans were acquired with 512 × 128 macular cube and 5-line high-definition raster scan protocols [13].

### Evaluation of GA progression

**Fig. 1:**
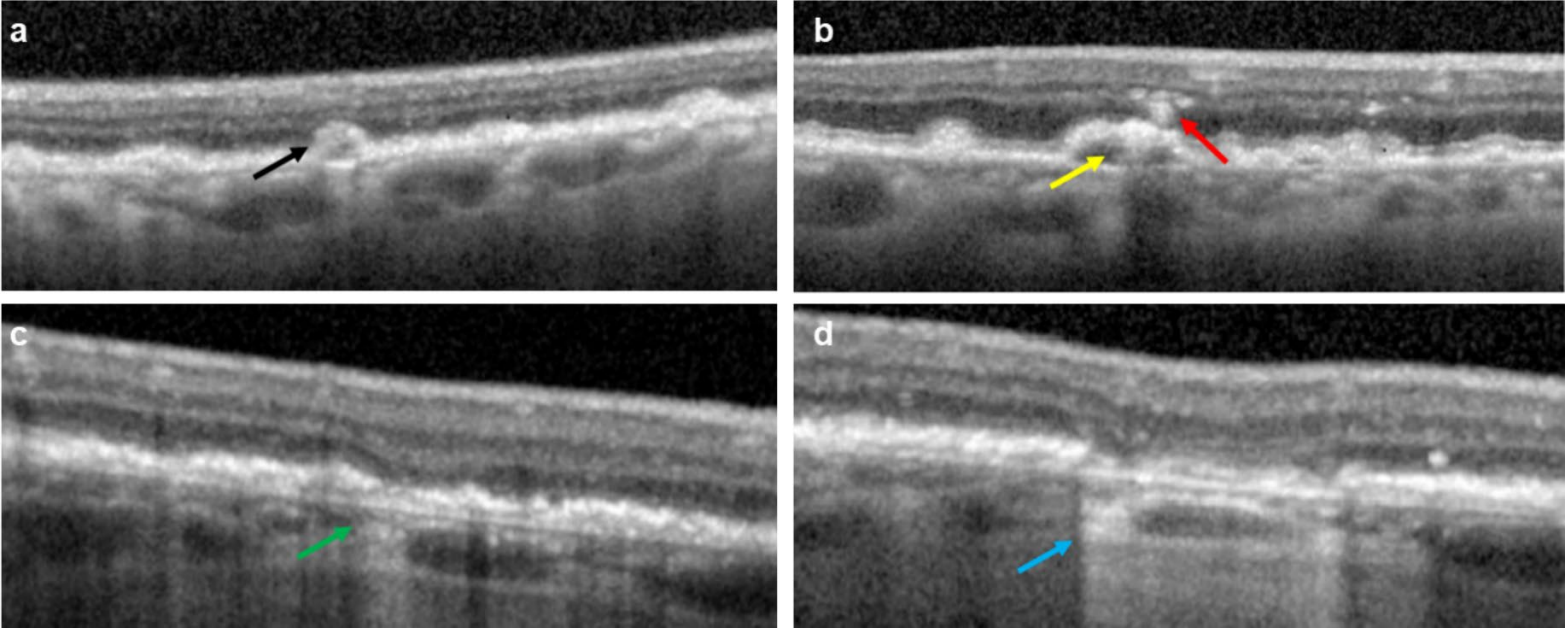
Representative images of OCT features of interest. (**a**) Larger drusen ≥ 40 µm in height (black arrow), (**b**) drusen with hyporeflective core (yellow arrow) and intraretinal hyperreflective foci (red arrow), (**c**) incomplete retinal pigment epithelium and outer retinal atrophy (iRORA, green arrow), and (**d**) conversion to complete retinal pigment epithelium and outer retinal atrophy at a follow-up visit (cRORA, blue arrow)

For this post hoc analysis, OCT data were securely transferred to the Doheny Image Reading and Research Lab for masked analysis. To identify drusen or iRORA for longitudinal assessment, regions of OCT volume scans more than 500 μm from the border of GA lesion(s) were evaluated by masked readers with experience in grading the progression of iRORA to cRORA and the progression of drusen to iRORA or cRORA based on CAM criteria at baseline and at months 6, 12, and 18 (Fig. 1) [15, 16]. Drusen of at least 40 µm in height were selected as an inclusion criterion because previous studies suggested that taller drusen are more likely to progress to atrophy and because these drusen are clearly identifiable [20, 21].

### Grading protocol for iRORA and large drusen

iRORA lesions and large drusen with height > 40 µm present at baseline and located ≥ 500 µm from the GA border were included. The longitudinal assessment was based on selecting a maximum number of 5 iRORA lesions and 5 drusen > 40 µm. In eyes with more than 5 lesions present, the selection was performed at baseline (masked to subsequent visits) based on which lesions (iRORA or drusen) were most definitive and clearly resolved on the OCT B scans. At baseline we assessed the number of iRORA lesions and large drusen (> 40 µm in height) per eye using an ordinal scale (1, 2, 3, 4, 5, > 5, not present). Only the height of the tallest drusen was reported. Thus, we assessed the progression of iRORA lesions and drusen to atrophy at the “lesion” level.

### Statistical analysis

Baseline characteristics were summarized using descriptive statistics. Progression was summarized at months 6, 12, and 18 using descriptive statistics (counts and percentages).

## Results

### Baseline characteristics

**Fig. 2:**
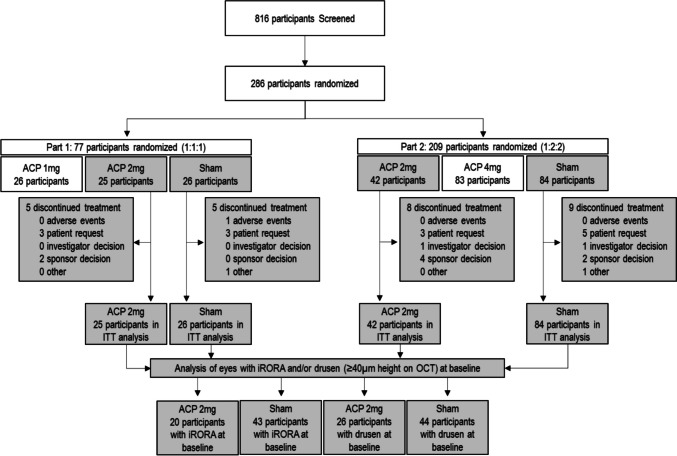
Participant flow diagram. Participants included in post hoc analysis of GATHER1 [13] are shaded in gray. ACP, avacincaptad pegol; iRORA, incomplete retinal pigment epithelium and outer retinal atrophy; ITT, intent-to-treat; OCT, optical coherence tomography

In GATHER1, 67 participants were randomized and treated with ACP 2 mg and 110 participants were randomized and treated with sham and included in the ITT analysis [13]. Before treatment, the number of iRORA lesions per eye using an ordinal scale (1, 2, 3, 4, 5, > 5) was evaluated. In the ACP 2 mg group, 8, 7, 2, 3, 0, and 4 eyes had 1, 2, 3, 4, 5, and > 5 iRORA lesions, respectively. In the sham group, 22, 9, 2, 5, 1, and 6 eyes had 1, 2, 3, 4, 5, and > 5 iRORA lesions, respectively. The same approach was used for the assessment of baseline drusen (> 40 µm in height). In the ACP 2 mg group, 9, 4, 3, 1, 1, and 11 eyes had 1, 2, 3, 4, 5, and > 5 drusen > 40 µm in height, respectively, and in the sham group, 11, 6, 4, 4, 1, and 22 eyes had 1, 2, 3, 4, 5, and > 5 drusen, respectively. Of the drusen > 40 µm in height, the mean height was 74.9 µm ± 22.6 and 79.8 µm ± 27.7 at baseline for ACP 2 mg and sham groups, respectively. In this post hoc analysis of GATHER1, participants were included if they received at least one dose of ACP 2 mg or sham, had iRORA or drusen of at least 40 μm in height at baseline, had gradable baseline and follow-up images, and did not develop MNV during the study (Fig. 2). At baseline, iRORA was present in 20 (29.9%) and 43 (39.1%) of eyes in the ACP 2 mg and sham groups, respectively. Drusen with a height of at least 40 µm were present in 26 (38.8%) eyes and 44 (40.0%) eyes in the ACP 2 mg and sham groups, respectively. Demographic and baseline characteristics were balanced across treatment arms and are summarized in Table 1.

**Table 1:** Demographics and baseline characteristics in eyes with iRORA or drusen at baseline

Demographics and baseline characteristics	iRORA at baseline		Drusen at baseline	
	ACP 2 mg (*n* = 20)	Sham (*n* = 43)	ACP 2 mg (*n* = 26)	Sham (*n* = 44)
Age, mean, y	76.3	79.1	76.7	76.6
Female gender, *n* (%)	10 (50.0)	29 (67.4)	14 (53.8)	29 (65.9)
Active smoker, *n* (%)	8 (40.0)	16 (37.2)	10 (38.5)	14 (31.8)
Non-subfoveal GA, *n* (%)	20 (100.0)	40 (93.0)	24 (92.3)	40 (90.9)
GA area, mean, mm^2^	6.87	7.48	6.99	7.74
GA area, mean square root, mm	2.55	2.64	2.57	2.70
Bilateral GA, *n* (%)	20 (100.0)	41 (95.3)	26 (100.0)	43 (97.7)
Hyperautofluorescence, *n* (%)	19 (95.0)	42 (97.7)	25 (96.2)	43 (97.7)
BCVA, mean, ETDRS letters	67.9	68.3	72.7	71.0
LL-BCVA, mean, ETDRS letters	38.0	36.2	45.2	39.5
LLD	31.7	32.0	27.6	31.5

*ACP* Avacincaptad pegol; *BCVA* Best corrected visual acuity; *ETDRS* Early Treatment Diabetic Retinopathy Study; *GA* Geographic atrophy; *iRORA* Incomplete retinal pigment epithelium and outer retinal atrophy; *LL-BCVA* Low-luminance BCVA; *LLD* Low-luminance deficit

### iRORA to cRORA progression

**Fig. 3:**
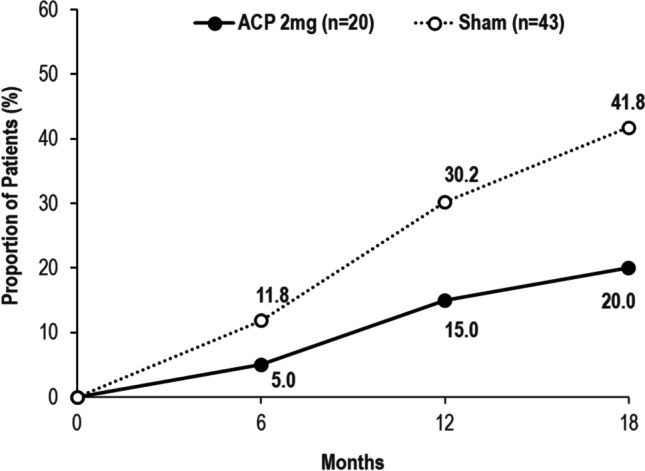
Proportion of eyes progressing from iRORA to cRORA (ACP 2 mg vs. sham). ACP, avacincaptad pegol; cRORA, complete retinal pigment epithelium and outer retinal atrophy; iRORA, incomplete retinal pigment epithelium and outer retinal atrophy

The proportion of eyes progressing from iRORA to cRORA in the ACP 2 mg group was 5.0%, 15.0%, and 20.0% at months 6, 12, and 18 vs. 11.8%, 30.2%, and 41.8% of sham-treated eyes, respectively. The absolute difference from sham was 6.8%, 15.2%, and 21.8% at 6, 12, and 18 months (Fig. 3).

**Fig. 4:**
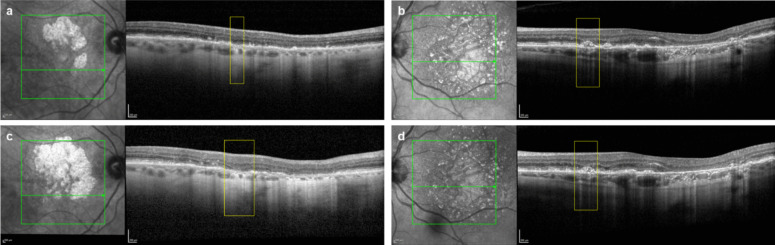
Representative masked examples of (**a**, **b**) eyes with iRORA at baseline that (**c**) progressed to cRORA, and (**d**) did not progress to cRORA over 18 months. cRORA, complete retinal pigment epithelium and outer retinal atrophy; iRORA, incomplete retinal pigment epithelium and outer retinal atrophy

Figure 4 illustrates masked examples of eyes with iRORA at baseline that did and did not progress to cRORA over 18 months.

### Drusen to iRORA/cRORA progression

**Fig. 5:**
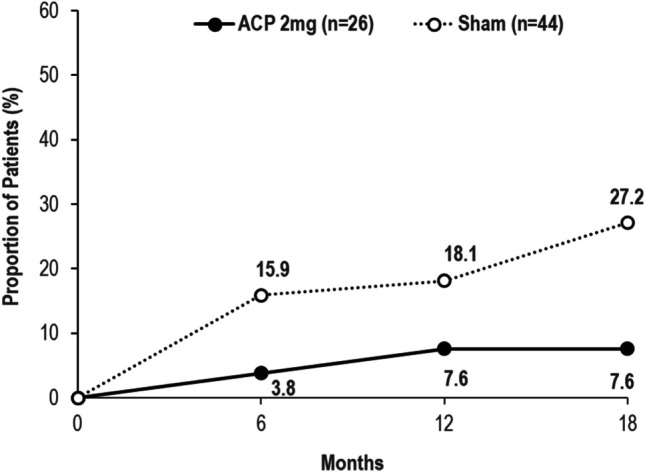
Proportion of eyes progressing from drusen to iRORA/cRORA (ACP 2 mg vs. sham). ACP, avacincaptad pegol; cRORA, complete retinal pigment epithelium and outer retinal atrophy; iRORA, incomplete retinal pigment epithelium and outer retinal atrophy

**Fig. 6:**
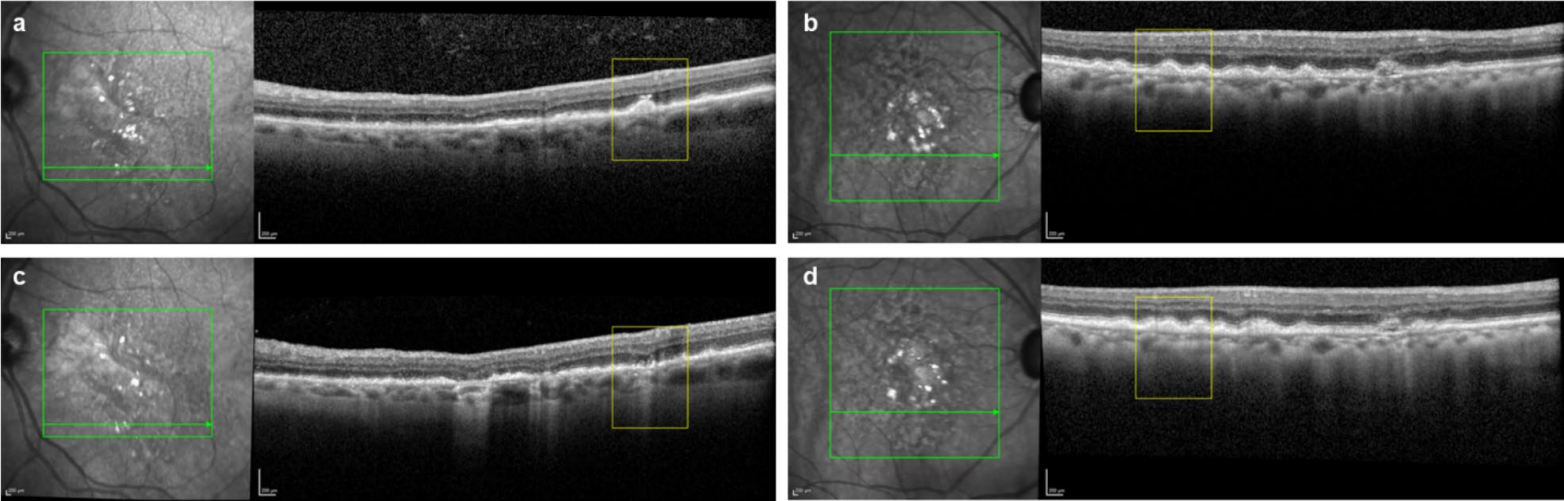
Representative masked examples of (**a**, **b**) eyes with drusen at baseline that (**c**) progressed to iRORA or cRORA, and (**d**) did not progress to iRORA or cRORA over 18 months. cRORA, complete retinal pigment epithelium and outer retinal atrophy; iRORA, incomplete retinal pigment epithelium and outer retinal atrophy

The proportion of eyes progressing from drusen to iRORA or cRORA in the ACP 2 mg group was 3.8%, 7.6%, and 7.6% at months 6, 12, and 18 vs. 15.9%, 18.1%, and 27.2% of sham-treated eyes, respectively. The absolute difference from sham was 12.1%, 10.5%, and 19.6% at 6, 12, and 18 months (Fig. 5). Examples of eyes with drusen at baseline that did or did not progress to iRORA or cRORA are shown in Fig. 6.

## Discussion

This post hoc analysis of GATHER1 evaluated progression of iRORA to cRORA and progression of drusen (≥ 40 µm in height) to iRORA/cRORA based on CAM criteria for atrophy classification. In patients treated with ACP 2 mg vs. sham, lower proportions of eyes progressing from drusen to iRORA/cRORA and from iRORA to cRORA were observed. These findings suggest a potential role for ACP in delaying progression of existing pre-atrophic AMD lesions. Furthermore, the results support the sustained reduction in GA lesion growth after 18-month treatment with ACP 2 mg. In the original prespecified analysis, the least squares mean change from baseline to Month 18 in square-root GA lesion area was 0.430 mm in ACP 2 mg-treated patients vs. 0.599 mm in sham-treated patients (28.1% reduction; *p* < 0.0014; *p*-values descriptive in nature), demonstrating a greater reduction in GA lesion growth, which continued from the 12-month time point [13, 14].

The ability to identify atrophy and atrophic precursors on OCT allows for evaluation of progression to GA and highlights an opportunity for early intervention to slow AMD progression. The reduction in progression from drusen to iRORA or cRORA was seen as early as 6 months with ACP 2 mg, and the separation between groups increased over time. These results suggest that treatment with ACP 2 mg may slow progression of drusen to iRORA or cRORA. Indeed, complement pathway components, including C5, have been identified in drusen, and the results from this analysis further support a role for the complement pathway in GA pathogenesis [22–24]. Immunohistochemical evidence has demonstrated an upregulation of complement membrane attack complex (MAC) in eyes with drusen, as well as significantly higher MAC levels in RPE-choroid samples of eyes with AMD vs. those of age-matched controls. Thus, ACP, which binds and inhibits C5, inhibits MAC by preventing the production of terminal, active C5 cleavage products (C5a and C5b), regardless of the initiating pathway [25, 26]. This may potentially pose a hypothesis of how ACP slows the progression of GA. Together, these results suggest that a greater treatment effect may be observed with earlier ACP 2 mg intervention, potentially protecting photoreceptors and preserving visual function.

In a natural history study of iRORA to cRORA progression, 27.5% of iRORA lesions progressed to cRORA within 12 months and 65.5% progressed from 12 to 24 months, resulting in a total of roughly 93% progressing within 24 months [27]. A similar pattern was seen in the sham group in this post hoc analysis of GATHER1, whereas the ACP 2 mg group showed reduced progression of iRORA to cRORA. These findings are compatible with the results of a post hoc analysis of the FILLY study, which demonstrated that areas of iRORA outside the GA area progressed to GA at a lower rate among those who received pegcetacoplan monthly or every other month compared with sham [28]. This further supports the relevance of the complement system in the complex pathophysiology of AMD.

The post hoc nature of this analysis, in which the hypothesis for lesion progression was generated using GATHER1 data, is a key limitation. Furthermore, because of the requirement for gradable baseline and follow-up images and the presence of iRORA and/or drusen of at least 40 μm in height at baseline, the sample size was relatively small. However, the main strengths of the study include the high-quality source data from a prospective, randomized, double-masked study, as well as analysis by masked readers experienced with the CAM grading criteria.

Overall, the findings of this post hoc analysis of GATHER1 suggest a potential effect of ACP 2 mg treatment in the earlier stages of AMD prior to the progression to GA, characterized by the CAM criteria. Our findings also provide further support for the role of complement pathway overactivation in the progression of AMD. In light of these findings, additional prospective studies are warranted.
